# Construction of Electrospun ZnO-NiO Nanofibers for Enhanced Ethanol Gas Sensing

**DOI:** 10.3390/s24237450

**Published:** 2024-11-22

**Authors:** Maryam Bonyani, Seyed Mojtaba Zebarjad, Tae-Un Kim, Hyoun Woo Kim, Sang Sub Kim

**Affiliations:** 1 Department of Materials Science and Engineering, Shiraz University, Shiraz 71454, Iran; maryambonyani@hafez.shirazu.ac.ir; 2 Department of Materials Science and Engineering, Inha University, Incheon 22212, Republic of Korea; xodjs635@naver.com; 3 The Research Institute of Industrial Science, Hanyang University, Seoul 04763, Republic of Korea; hyounwoo@hanyang.ac.kr; 4 Division of Materials Science and Engineering, Hanyang University, Seoul 04763, Republic of Korea

**Keywords:** electrospinning, ZnO, NiO, ethanol, gas sensor, sensing mechanism

## Abstract

Semiconducting metal oxides with nanofiber (NF) morphologies are among the most promising materials for the realization of gas sensors. In this study, we have prepared electrospun ZnO-NiO composite NFs with different amounts of NiO (0, 20, 40, 60 and 80% wt%) for the systematic study of ethanol gas sensing. The fabricated composite NFs were annealed at 600 °C for crystallization. Based on characterization studies, NFs were produced with desired morphologies, phases, and chemical compositions. Ethanol gas sensing studies revealed that the sensor with 40 wt% NiO had the highest response (3.6 to 10 ppm ethanol) at 300 °C among all gas sensors. The enhanced gas response was ascribed to the formation of sufficient amounts of p-n NiO-ZnO heterojunctions, NFs’ high surface areas due to their one-dimensional morphologies, and acid–base interactions between ZnO and ethanol. This research highlights the need for the optimization of ZnO-NiO composite NFs so that they achieve the highest sensing response, which can be extended to other similar metal oxides.

## 1. Introduction

Owing to rapid industrialization and urbanization, volatile organic compounds (VOCs) are being continuously emitted into the air from various processes and sources like waste water treatment, petrochemical processes, petroleum refining, and so on [1,2,3,4]. Among VOCs, ethanol (C_2_H_5_OH) is a common industrial raw material and solvent, being especially prevalent in the food and chemical industries [5,6]. However, exposure to ethanol can result in a headache, drowsiness, and eye irritation [7,8]. Besides, alcohol consumption is a leading factor in vehicle accidents worldwide. Hence, the detection of ethanol is very important from industrial and safety viewpoints [9,10].

Among various gas sensors, resistive ones are highly popular due to their high response, rapid dynamics, small size, excellent stability, and low price [11,12]. Nonetheless, they often need high temperatures to obtain optimal performances, and they generally have poor selectivity [13].

Semiconducting n-type ZnO, which has high thermochemical stability, a wide energy gap (3.37 eV), and high charge carrier mobility, is utilized for fabricating resistive sensors for the detection of toxic gasses [14,15,16]. However, its performance in a pristine form is not high, and therefore, approaches such as doping, noble metal loading, and p-n heterojunction formation [17] are used to boost its sensing performance. In particular, in p-n heterojunctions, there are several sources of resistance modulation. Hence, the p-n heterojunction formation approach is highly popular. NiO is a well-known p-type metal oxide. It has a bandgap of 3.5 eV and is widely used for the sensing of gasses [18,19,20,21]. Accordingly, the formation of ZnO-NiO heterojunctions is a feasible method of achieving high gas sensing features. For instance, the enhanced acetone sensing features of ZnO-NiO composites, relative to those of pristine NiO and ZnO sensors, were shown by Kavitha et al. [22] and Liu et al. [23]. Another study found that a ZnO-NiO heterojunction sensor could detect 500 ppm ethanol with a response of 8.1 at 125 **°C** under UV light conditions [24]. Although there are papers on the gas sensing capabilities of ZnO-NiO composites [25,26], the ethanol sensing characteristics of ZnO-NiO composite nanofibers (NF) gas sensors have not been studied. NFs can be easily synthesized via an electrospinning method, and owing to their extensive surface areas, they may provide numerous adsorption sites for gas molecules [27,28].

In view of the lack of a systematic study on ZnO-NiO composite NFs, in this work, we fabricated ZnO-NiO composite NFs with different NiO contents (0, 20, 40, 60 and 80 wt%) and explored their ethanol gas sensing features. The fabricated NFs were characterized using advanced techniques, and gas sensors were then fabricated. Among the fabricated sensors, the one containing 40 wt% NiO showed a superior response to ethanol at 300 °C, attributable to the generation of a sufficient number of p-n heterojunctions, the existence of oxygen vacancies, and the sensor’s high surface area.

## 2. Materials and Methods

### 2.1. Preparation of ZnO-NiO Composite NFs

We obtained zinc acetate [Zn((CH_3_CO_2_)_2_)], nickel(II) nitrate hexahydrate (Ni (NO_3_)_2_.6H_2_O), and polyvinyl alcohol (PVA; MW ~80,000) of analytical grades from Merck. First, PVA was dissolved in deionized water to obtain an aqueous PVA (10 wt.%) solution. Subsequently, a solution of 7% wt. powder containing specific amounts of zinc acetate and different amounts of nickel nitrate was added to the solution, which was then stirred at 70 °C for 2h to achieve a viscous consistency. Table 1 provides detailed information on the specific quantities of the various ZnO-NiO composite NFs used. The viscous solution was drawn into a plastic syringe (1.13 mm in diameter), which was then attached to an anode linked to a high-voltage power supply. A voltage of 16 kV was applied to a needle positioned 14 cm away from the rotating collector. Under the application of a high voltage and a constant feeding rate of 0.5 mL/h, a Taylor cone was produced, and electrospun ZnO-NiO NFs accumulated on the Al collector. To completely remove the polymeric species, we annealed the composite NFs at 600 °C for 2 h. Pristine ZnO NFs were also produced using the same procedure, but without adding nickel nitrate.

### 2.2. Characterizations

Field emission scanning electron microscopy (FE-SEM; Hitachi S-4200, Tokyo, Japan) and transmission electron microscopy (TEM; JEOL; Tokyo, Japan) were employed to examine the morphologies of the synthesized NFs, and energy-dispersive X-ray spectroscopy (EDS) incorporated in TEM was used to find their compositions. The phase of the NFs was determined using X-ray diffraction (XRD; Philips X’Pert, Almelo, The Netherlands) with Cu K_α_ radiation (λ = 1.5418 Å). The surface chemical states of the NFs were ascertained using X-ray photoelectron spectroscopy (XPS; Thermo Scientific, Waltham, MA, USA).

### 2.3. Gas Sensing Measurements

**Scheme 1:**
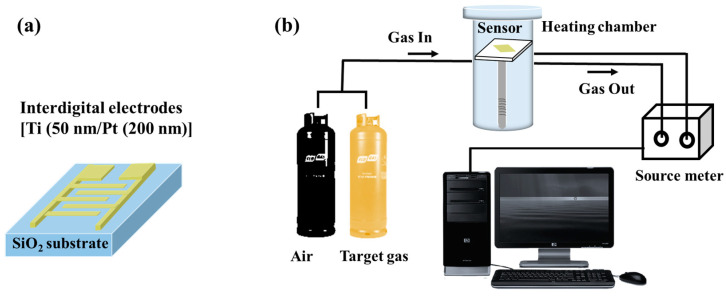
Schematic illustration of (**a**) gas sensor substrate and (**b**) the gas sensing test system.

The sensing materials were mixed in α-terpineol (10 μL), and they were drop-coated onto the SiO_2_ substrate equipped with Ti (50 nm)/Pt (200 nm) bi-layer electrodes (Scheme 1a). We used α-terpineol for this purpose due to its non-toxic and environmentally friendly nature. Furthermore, it can be evaporated easily, leaving dry sensing material on the surface of substrate. Then, the prepared sensors were conditioned at 300 °C for 2 h. They were then put in a gas chamber in a horizontal tubular quartz furnace. The required quantities of gasses were delivered into the gas chamber via precise mass flow controllers. The total gas flow rate was set to 500 sccm. The resistances of the sensors were recorded in air (*R*_a_) and in the target gas atmosphere (*R*_g_) and, depending on the gas sensor and gas type, the gas response was obtained as *R* = *R*_a_/*R*_g_ or *R*_g_/*R*_a_. Scheme 1b shows the gas sensing measurement system. The response time was calculated as the time required for the sensor’s resistance to reach to its 90% final value in the presence of ethanol and recovery time was calculated as the time required for the sensor resistance’s to reach to 90% of its initial value after the stoppage of ethanol gas.

## 3. Results and Discussion

### 3.1. Morphological and Structural Investigations

**Figure 1:**
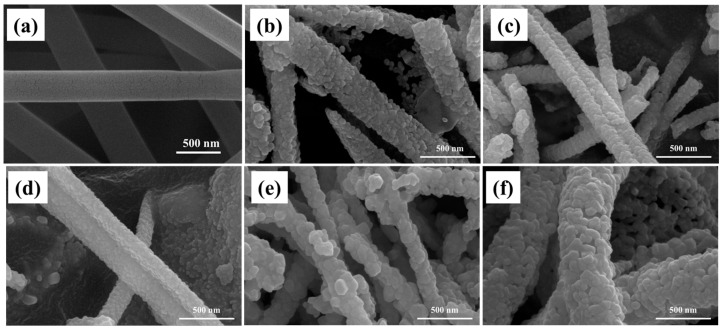
SEM micrographs of (**a**) pristine ZnO NFs before annealing and (**b**) after annealing. SEM images of ZnO-NiO composite NFs with (**c**) 20, (**d**) 40, and (**e**) 60 and (**f**) 80 wt.% NiO after annealing.

Figure 1a shows SEM morphology of pristine ZnO NFs before annealing. As shown, the surface is quite smooth, with a diameter of approximately 400 nm. However, after annealing, due to the evaporation of organic species, the surface becomes rough and the diameter decreases slightly (Figure 1b). It should be noted that the final diameter of NFs depends on various parameters such as applied voltage, the distance from the nozzle to the collector, the annealing temperature, and so on. Figure 1b–f show FE-SEM views of the ZnO-NiO composite NFs with different NiO contents after annealing. Their surfaces are rough, and their diameters are ~150–300 nm. Due to the evaporation of the solvent and the organic species during annealing, the surfaces of the NFs were rough and contained ultrafine grains. Overall, all samples had very similar morphologies since they were synthesized using the same procedure.

**Figure 2:**
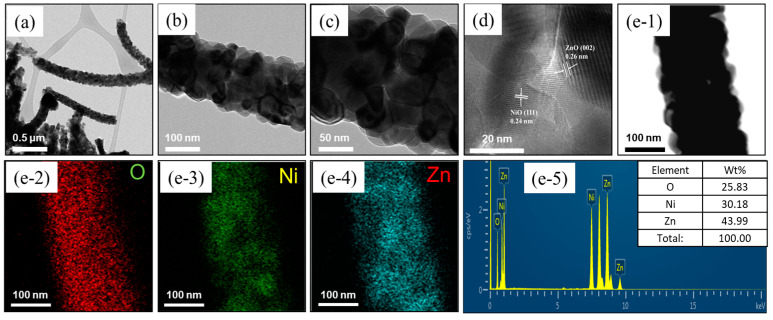
(**a**–**c**) TEM (**d**) HRTEM image (**e-1**–**e-5**) EDS analysis of ZnO-NiO (40 wt%) composite NFs.

**Figure 3:**
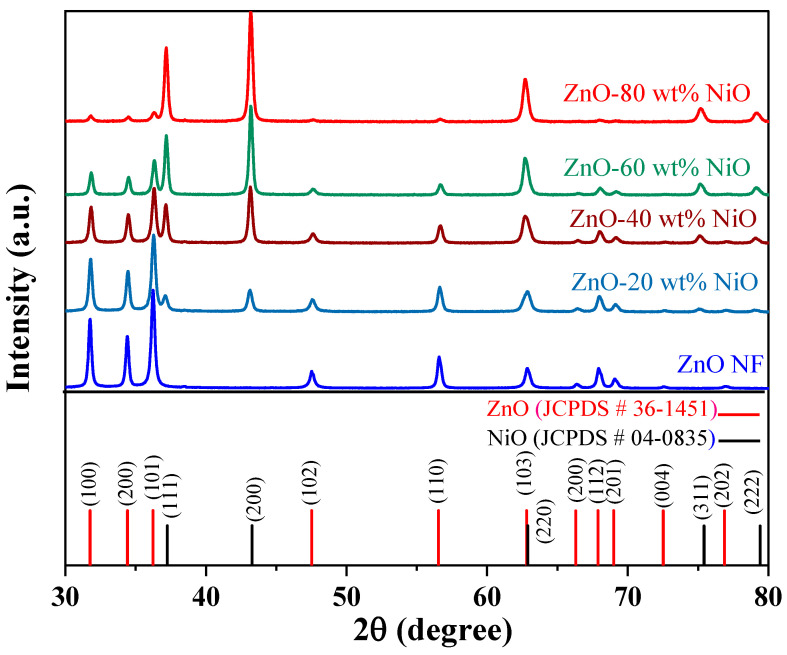
XRD patterns of pristine ZnO NFs and ZnO-NiO composite NFs with different NiO contents.

**Figure 4:**
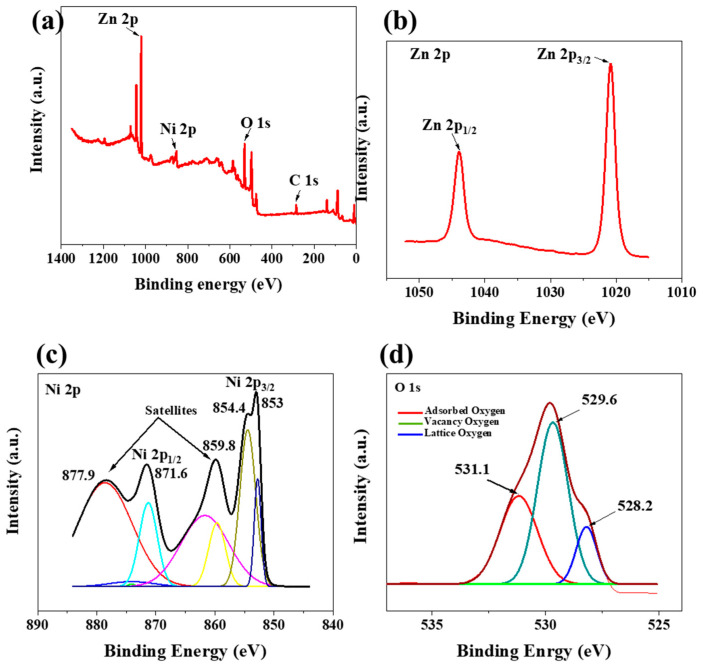
(**a**) XPS survey scan of ZnO-NiO (40 wt%) composite NFs, and XPS core-level spectra of (**b**) Zn 2p, (**c**) Ni 2p, and (**d**) O 1s.

**Figure 5:**
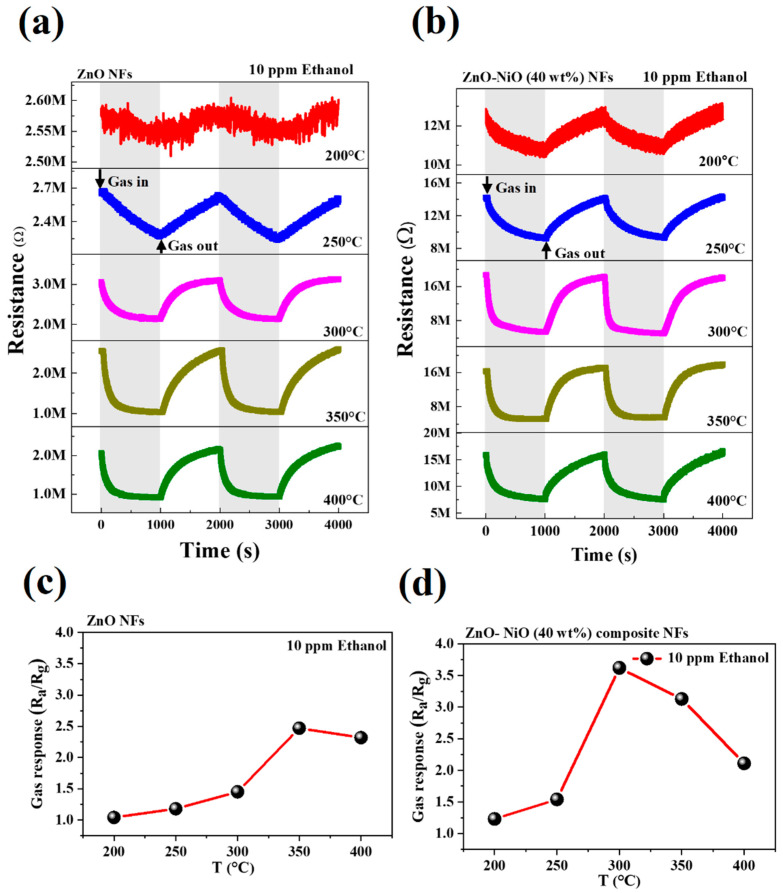
Dynamic resistance curves of (**a**) pristine ZnO and (**b**) ZnO-NiO (40 wt%) composite NF gas sensors for 10 ppm ethanol gas at various temperatures. A comparison of the response versus temperature for (**c**) pristine ZnO and (**d**) ZnO-NiO (40 wt%) composite NFs gas sensors for 10 ppm ethanol.

Figure 2a–c present TEM images of the ZnO-NiO (40 wt%) NF heterostructures. They have diameters in the range of 150–300 nm, and have rough surfaces displaying nanograins. The HRTEM image reveals the fringes, with interplanar distances of 0.24 and 0.26 nm. These values are related to the interplanar spacing of the (002) planes of hexagonal ZnO and the (111) planes of crystalline NiO (Figure 2d). The EDS mapping results of the ZnO-NiO (40 wt%) NFs are presented in Figure 2(e-1)–(e-4), with Figure 2(e-1) showing the presence and distribution of Zn, Ni, and O elements within captured NFs. Based on HRTEM images and the overlapping distribution of both Zn and Ni elements, it can be concluded that ZnO-NiO heterojunctions exist in composite NFs. Figure 2(e-5) shows the EDS spectrum of ZnO-NiO (40 wt%) composite NFs, in which the weight percentages of O, Ni, and Zn elements were 25.83, 30.18, and 43.99 wt.%.

Figure 3 shows the XRD patterns of pristine ZnO and ZnO-NiO composite NFs with various NiO content. For pristine ZnO NFs, all diffraction peaks were related to the crystal planes of hexagonal wurtzite ZnO (JCPDS No. 36-1451). For composite NFs, new peaks appeared at Bragg angles of 37.18°, 43.1°, 62.88°, 75.24°, and 79.23°, and they corresponded to the (111), (200), (220), (311), and (222) crystal planes of the cubic phase of NiO (JCPDS No. 73-1523). Since the NiO content in composite NFs rose, the intensity of NiO peaks increased gradually, reflecting the higher amount of NiO. The co-existence of NiO and ZnO phases is beneficial for sensing applications as they form p–n heterojunctions [29,30].

Figure S1a exhibits the XPS survey scan of pristine ZnO NFs. Peaks associated with the elements Zn, O, and C (from the surrounding environment) are clearly visible. Figure S1b displays the Zn 2p core-level region, featuring two primary peaks attributed to Zn 2p_3/2_ and Zn 2p_1/2_ at 1021.5 and 1044.5 eV, respectively; these peaks can be related to the presence of Zn^2+^ ions in ZnO. The O 1s core-level region (Figure S1c) is deconvoluted into two main peaks at 529.7 and 531.2 eV, which belong to lattice oxygen and adsorbed oxygen species, respectively.

Figure 4a depicts the XPS survey of ZnO-NiO (40 wt%) composite NFs, and peaks related to the Zn, Ni, O, and C (from the surrounding environment) can be observed. Notably, there are no peaks related to impurity elements. To obtain more insights, we examined the XPS core-level regions of different elements. Figure 4b displays the Zn 2p core-level region; similar to pristine ZnO, two peaks can be seen that are centered at 1021.5 and 1044.5 eV. These are related to Zn 2p_3/2_ and Zn2p_1/2_, respectively and they are connected to the existence of Zn^2+^ in ZnO [14,29]. The Ni 2p core-level region comprising five peaks is displayed in Figure 4c. The peaks at 853, 854.4, and 859.8 eV are assigned to Ni 2p_3/2_ and its satellite peak, and the two peaks at 871.6 and 877.9 eV are attributed to Ni 2p_1/2_ and its satellite peak [31]. In the Ni 2p_3/2_ region, the peak at 853 eV can be ascribed to Ni^2+^ ions, while that at 854.4 is related to Ni^3+^ ions. The two shake-up satellite peaks at 859.8 and 877.9 eV are indicative of NiO [32,33]. The O 1s core-level region was deconvoluted into three peaks at 528.2, 529.7, and 531.2 eV. These belonged to lattice oxygen, oxygen vacancy defects, and adsorbed oxygen species, respectively (Figure 4d) [29,34].

### 3.2. Gas Sensing Investigations

Figure 5a,b show the sensing curves of pristine ZnO and ZnO-NiO (40 wt%) composite NF sensors for 10 ppm ethanol gas at various temperatures, respectively. Both sensors exhibited n-type characteristics at all temperatures because the ZnO phase was dominant in them. For better comprehension, in Figure 5c,d, the response values of both pristine ZnO and ZnO-NiO (40 wt%) composite NFs gas sensors are plotted against the sensing temperature for 10 ppm ethanol gas, respectively. At every temperature, the composite sensor indicated a greater response compared to the pristine sensor. In particular, the optimal sensing temperature for the composite sensor was 300 °C. It was higher for the pristine sensor, standing at 350 °C; this showed the potential of composites to reduce the sensing temperature, similar to what was seen in a previous study [35].

**Figure 6:**
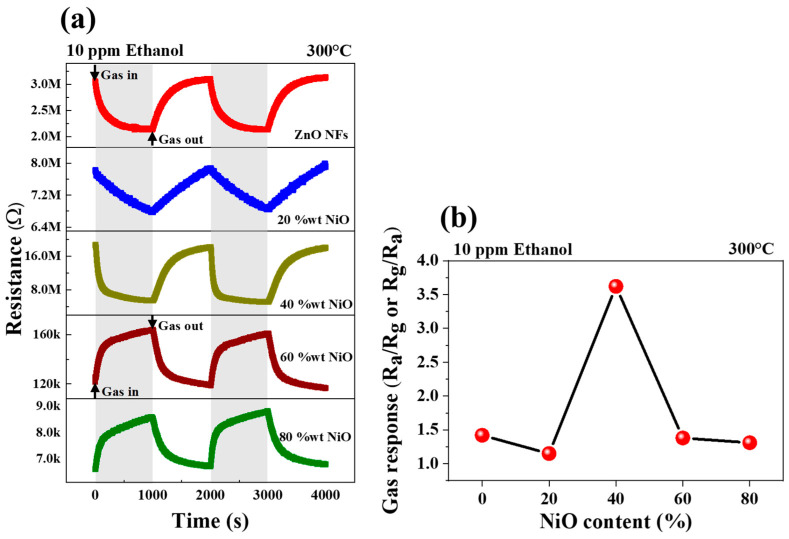
(**a**) Dynamic resistance plots of the ZnO-NiO composite NFs sensors with varying NiO contents (0–80 wt%) for 10 ppm ethanol gas at 300 °C. (**b**) The response of the composite gas sensors to 10 ppm ethanol versus NiO content.

Figure 6a shows the dynamic resistance curves of ZnO-NiO composite NFs with varying NiO contents for 10 ppm ethanol gas at 300°C. ZnO-rich composite NF sensors (NiO = 0, 20, and 40 wt%) showed an n-type response to ethanol gas. By contrast, NiO-rich gas sensors (NiO = 60 and 80 wt%) exhibited p-type characteristics, as their resistances increased upon exposure to ethanol gas. Thus, for ZnO-rich gas sensors, the ZnO phase, in which the dominant charge carriers were electrons, was the main component determining the sensing performance, while for NiO-rich gas sensors, the NiO phase, in which the basic charge carriers were holes, was crucial for the detection of ethanol gas. To obtain more insights, we plotted the response of the gas sensors to 10 ppm ethanol against the NiO content (Figure 6b). The responses were 1.4, 1.15, 3.7, 1.38, and 1.32 for NiO contents of 0, 20, 40, 60, and 80 wt%, respectively. Thus, the sensor containing 40 wt% NiO exhibited the most significant response to ethanol gas.

**Figure 7:**
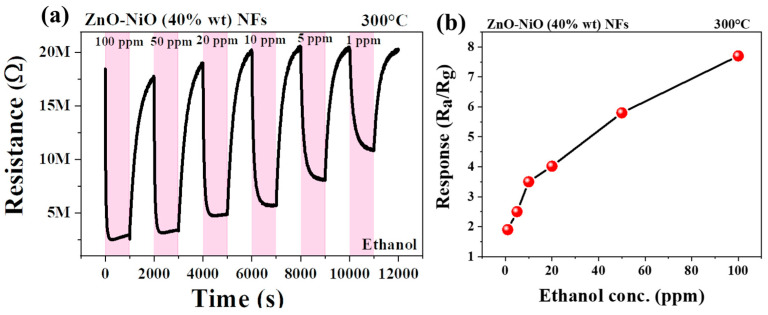
(**a**) Dynamic resistance plots of the ZnO-NiO composite NFs gas sensor with 40 wt% NiO for 100 ppm ethanol gas at 300 °C and (**b**) the corresponding calibration graph.

Figure 7a offers sensing graphs of the optimized sensor for various concentrations of ethanol gas at 300 °C. The sensor could detect all concentrations of ethanol. As indicated by the corresponding calibration curve (Figure 7b), the response to 1, 5, 10, 20, 50, and 100 ppm ethanol was 1.9, 2.5, 3.5, 4, 5.8, and 7.7, respectively. Thus, the sensor could detect both low and high concentrations of ethanol gas reliably. The response time and recovery time for optimal gas sensor to 10 ppm ethanol at 300 °C were calculated to be 77 and 118 s, respectively.

**Figure 8:**
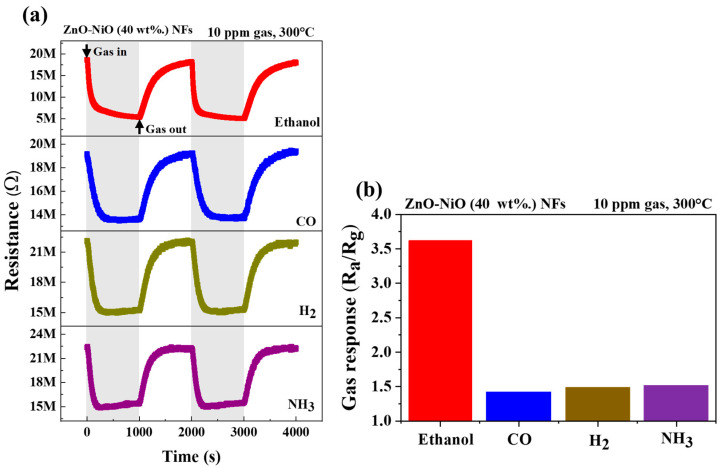
(**a**) Dynamic resistance plots of the ZnO-NiO composite NF sensor with 40 wt% NiO for a concentration of 10 ppm of different gasses at 300 °C and (**b**) the corresponding selectivity plot.

The ability to discriminate between different gasses is a key characteristic of an effective gas sensor for use in practical applications. Figure 8a presents selectivity graphs of the optimized sensor when subjected to 10 ppm of different gasses at 300 °C. The responses for the gasses H_2_, CO, NH_3_, and C_2_H_5_OH were 1.6, 1.4, 1.4, and 3.6, respectively (Figure 8b). Hence, the sensor manifested good selectivity toward ethanol, which is very important for its practical use.

**Figure 9:**
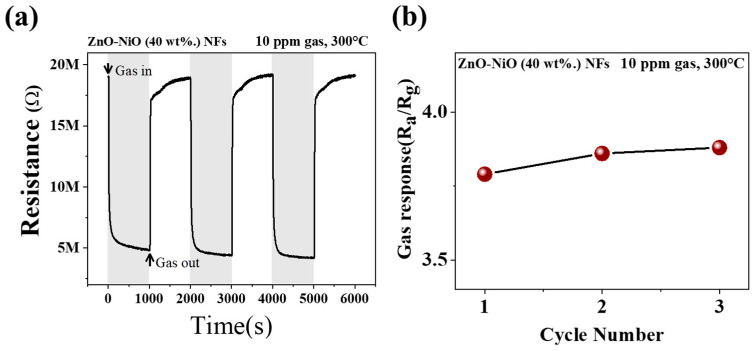
(**a**) Long-term stability (after six months) and repeatability (during three cycles) of ZnO-NiO (40 wt.%) NFs sensor to 10 ppm ethanol gas at 300 °C. (**b**) Variations in sensor response during three sequential cycles.

To check the long-term stability of the optimal sensor, it was exposed to 10 ppm ethanol at 300 °C after six months and during three sequential cycles to check its repeatability (Figure 9a). As shown in Figure 9b, the sensor showed a response close to its response in a fresh state (~3.65). Furthermore, there was not a significant difference between the responses during three sequential cycles. Therefore, the optimal sensor not only had good long-term stability but also good repeatability.

### 3.3. Proposed Sensing Mechanism

The fundamental sensing mechanism of resistive gas sensors relies on the alteration of resistance induced by the presence of target gasses [36,37]. First, when a resistive sensor is in the air, the high electron affinity of oxygen leads to the capture of electrons, resulting in the formation of ionic oxygen species that are adsorbed on the sensing layer surface [10,29]:

O_2(g)_ → O_2(ads)_ (1)

O_2(ads)_ + e^−^→O_2_^−^ (2)

O^−^_2_ + e^−^→2O^−^ (3)

O^−^ + e^−^ →O^2−^ (4)

Accordingly, in n-type gas sensors such as ZnO, an electron depletion layer (EDL) appears on the ZnO surface, and the electron concentration in the layer is much lower than that in the interior of the sensor. Hence, the sensor shows high resistance in air. In an ethanol atmosphere, ethanol interacts with pre-adsorbed oxygen ions, releasing electrons to the sensor’s surface, as shown below [10]:

C_2_H_5_OH _(ads)_ + 6O^−^ _(ads)_ → 2CO_2_ + 3H_2_O + 6e^−^ (5)

**Figure 10:**
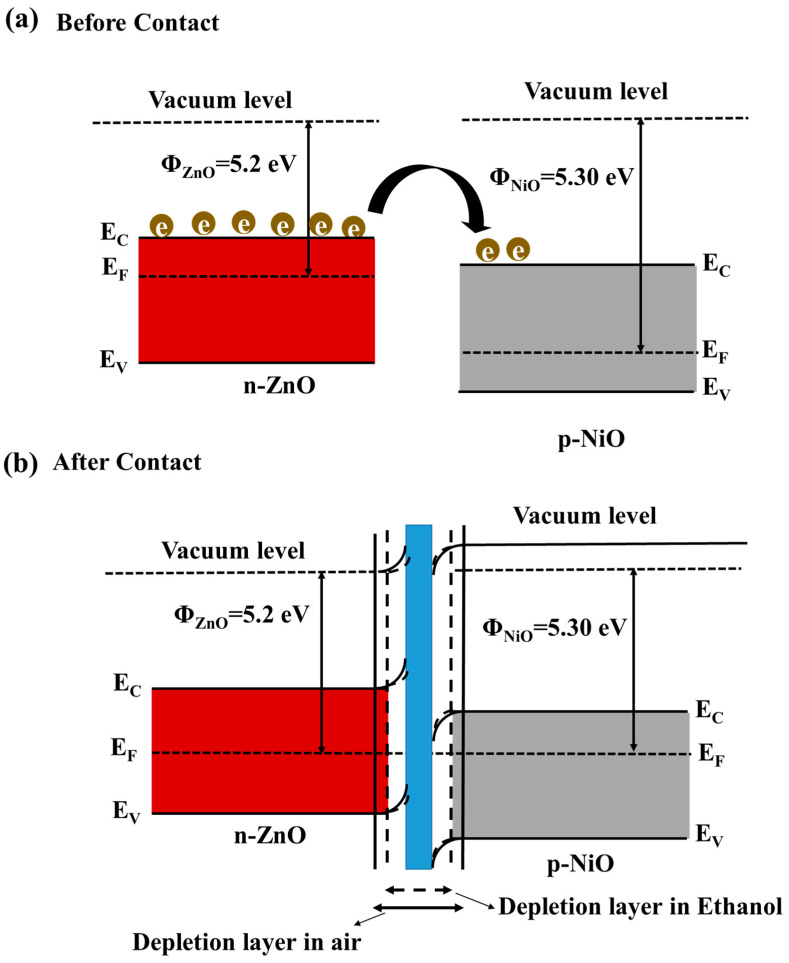
Band energy levels of ZnO and NiO (**a**) before and (**b**) after contact in air and ethanol.

The released electrons reduce the EDL’s thickness, causing the sensor’s electrical resistance to decrease in an ethanol atmosphere. However, the pristine ZnO sensor displays a relatively low response to ethanol gas. In composite NF gas sensors, the generation of p-n heterojunctions between n-ZnO and p-NiO should be considered [38]. Owing to the difference between the work functions (Φ) of ZnO (Φ = 5.2 eV) [36,39] and NiO (Φ = 5.3 eV) [40] (Figure 10a), when the two materials are in close contact, electrons migrate from ZnO to NiO, while holes flow in the opposite direction to balance the Fermi levels. This leads to band bending and the generation of heterojunctions in the contact regions in air (Figure 10b). Furthermore, owing to the flow of electrons to NiO, the thickness of the EDL on ZnO increases relative to that on the pristine ZnO sensor. This is evident in Figure 5a,b, where the baseline resistance of composite NF sensor is much higher than that of the pristine ZnO NF sensor at the same temperature. When the sensor is exposed to ethanol gas, the released electrons cause the significant thinning of the EDL and considerably decrease the height of potential barriers (Figure 10b). Hence, the resulting resistance modulation contributes to the generation of a sensing signal. The sensor with 20 wt% NiO exhibited a lower response compared to the pristine ZnO sensor. Thus, despite the presence of p-n heterojunctions in the composite NF sensor, it still showed a lower response than the pristine ZnO sensor. This may be related to the intrinsically higher sensing performance of ZnO compared with NiO. It has previously been reported that, for given morphological features, the response of p-type gas sensors is equivalent to the square root of the response of n-type gas sensors. Accordingly, it appears that despite the presence of p-n heterojunctions, some ZnO was replaced with NiO with poorer sensing properties, eventually resulting in a lower sensing performance. Increasing the NiO content to 40 wt% in composite NFs significantly improved the sensor response. In fact, in the sensor with 40 wt% NiO (optimal sensor), an optimal number of heterojunctions was formed, which led to the emergence of numerous sources of resistance modulation. In the sensors with higher NiO contents, p-type NiO became the dominant phase and, as stated above, the intrinsically lower gas sensing properties of p-NiO relative to ZnO led to the sensors having poorer sensing properties.

On acidic oxide surfaces, ethanol is first dehydrated to ethylene (C_2_H_4_), while on basic oxide surfaces, ethanol is dehydrogenated to acetaldehyde (CH_3_CHO). The intermediate products are further oxidized to H_2_O and CO_2_ [41]. Since ethylene has higher stability than acetaldehyde, ethanol sensing is promoted on surfaces of basic oxides such as ZnO [42]. Thus, in ZnO-NiO with 40 wt% NiO, owing to the higher amount of ZnO relative to composites with higher NiO content, stronger interactions are expected between the sensor and ethanol gas, resulting in higher sensing for ethanol. Finally, owing to their one-dimensional (1D) morphologies, the synthesized composite NFs provided abundant adsorption sites for ethanol gas molecules. However, the contribution of the 1D morphology to the sensing properties would have been the same for all gas sensors since they were all synthesized under the same conditions.

In Table 2, the ethanol gas sensing features of the optimized ZnO-NiO composite NF sensor (with 40 wt% NiO) are compared with those of previously reported sensors. In general, the present sensor’s performance is comparable to that of the other sensors. In particular, its relatively high sensing temperature should be decreased by adopting strategies like noble metal decoration and by operating the sensor in the self-heating mode. The sensing performance can be further improved by the decoration of the sensor’s surface with noble metals. Therefore, in our future studies, we will investigate this strategy in order to enhance the sensing performance.

## 4. Conclusions

We synthesized electrospun ZnO-NiO composite NFs with different NiO contents (0, 20, 40, 60 and 80 wt%) and crystallized them at 600 °C for 2 h. Using advanced techniques like XRD, FE-SEM, and XPS, we verified that they had the desired phase, morphology, and composition. The sensor with 40 wt% NiO displayed a response of 3.6 to 10 ppm ethanol at 300 °C, which was greater than the responses of the other gas sensors. This high sensing performance was related to the high surface area associated with the 1D NF morphology, the creation of a sufficient number of p-n NiO-ZnO heterojunctions, and the strong interaction between ZnO and ethanol. The gas sensing performance may be further boosted through decoration with noble metals, and this should be investigated in future studies.

## Figures and Tables

**Table 1:** Detailed information on the specific quantities of various ZnO-NiO composite NFs.

Composition	Zinc Acetate (g)	Nickel Nitrate (g)
ZnO	0.63	0
80ZnO-20NiO	0.5	0.18
60ZnO-40NiO	0.37	0.36
40ZnO-60NiO	0.25	0.54
20ZnO-80NiO	0.13	0.72

**Table 2:** Comparison of ethanol gas sensing performances between the present optimized sensor and the sensors reported in the literature.

Sensing Materials	T (°C)	Conc. (ppm)	Response (*R*_a_/*R*_g_) or (*R*_g_/*R*_a_)	Ref.
CuO-ZnO nanorods	350	25	19.4	[43]
Au-decorated PbS-SnS_2_ nanocomposite	RT	400	87	[44]
Fe_2_O_3_/Co_3_O_4_ nanocomposite	300	100	10.86	[45]
La_2_O_3_-doped SnO_2_ nanowires	400	100	57.3	[46]
In_2_O_3_/ZnSnO_3_ nanocubes	250	100	14.9	[47]
ZnO-SnO_2_ NFs	300	5	4	[48]
ZnO-NiO composite NFs	300	10	3.6	Present work

## Data Availability

Data are contained within the article.

## Supplementary Materials

The following supporting information can be downloaded at: https://www.mdpi.com/article/10.3390/s24237450/s1, Figure S1: (a) XPS survey of pristine ZnO NFs. XPS core-level spectra of (b) Zn 2p and (c) O 1s.
