# Supplementary material for: Construction of Electrospun ZnO-NiO Nanofibers for Enhanced Ethanol Gas Sensing
Source: Sensors (Basel). 2024 Nov 22;24(23):7450. doi: 10.3390/s24237450 (PMC11644565; doi:10.3390/s24237450)
Supplement: Supplementary file 1 [file sensors-24-07450-s001.zip › sensors-3292867-supplementary.pdf]

Supplementary Materials

Construction of Electrospun ZnO-NiO Nanofibers for Enhanced Ethanol Gas Sensing

Maryam Bonyani [1], Seyed Mojtaba Zebarjad [1,*], Tae-Un Kim [2], Yujin Kim [2], Hyoun Woo Kim [3,4] and Sang Sub Kim [2,*]

[1] Department of Materials Science and Engineering, Shiraz University, Shiraz 71454, Iran; maryambonyani@hafez.shirazu.ac.ir

[2] Department of Materials Science and Engineering, Inha University, Incheon 22212, Republic of Korea;
xodjs635@naver.com (T.-U.K.); kyj200805@gmail.com (Y.K.)

[3] The Research Institute of Industrial Science, Hanyang University, Seoul 04763, Republic of Korea;
hyounwoo@hanyang.ac.kr

[4] Division of Materials Science and Engineering, Hanyang University, Seoul 04763, Republic of Korea

* Correspondence: mojtabazebarjad@shirazu.ac.ir (S.M.Z.); sangsub@inha.ac.kr (S.S.K.)

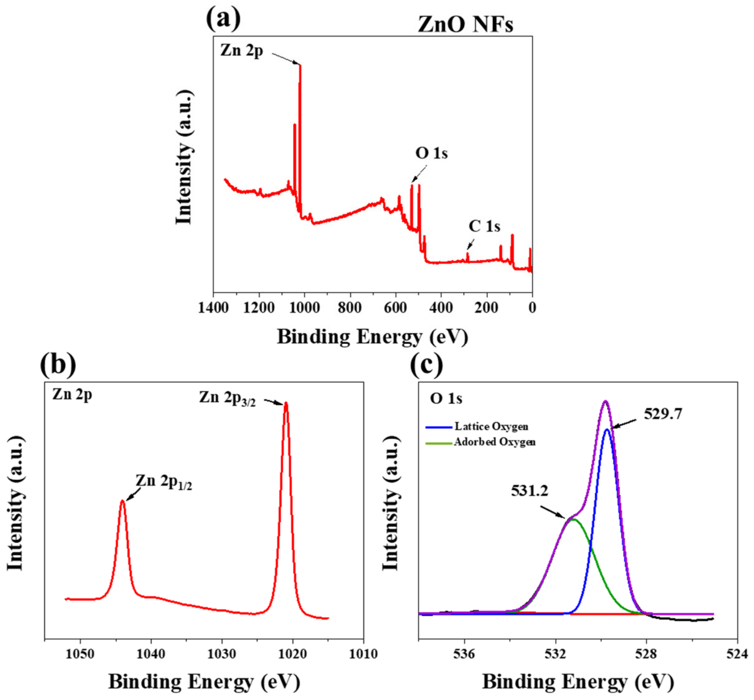

Figure S1. (a) XPS survey of pristine ZnO NFs. XPS core-level spectra of **(b)** Zn 2p and **(c)** O 1s.
